# Prognostic factors for intermediate- or high-risk neuroblastomas in children in China

**DOI:** 10.1186/s12887-023-04258-w

**Published:** 2023-12-06

**Authors:** Yi Zhang, Wei-Ling Zhang, Dong-Sheng Huang, Yi-Zhuo Wang, Hui-Min Hu, Tian Zhi, Yan-Yan Mei

**Affiliations:** Department of Pediatrics, Beijing Tongren Hospital, Capital Medical University, No 2, Xihuan South Road, Daxing Zone, Beijing, China

**Keywords:** Child, Neuroblastoma, Patients, Risk Assessment, Survival

## Abstract

**Background:**

Evidence regarding the characteristics and prognosis of neuroblastoma (NBL) in China is limited. We aimed to investigate the characteristics and prognosis of intermediate- or high-risk NBL in children in China.

**Methods:**

We included 147 patients with intermediate- or high-risk NBL evaluated from January 2006 to March 2015. The patients were aged 1 month to 15.5 years, 66% of them were boys, and 117 (79.6%) were diagnosed with high-risk NBL.

**Results:**

After a median follow-up of 32.5 months, 80 (45.6%) patients survived, with a median survival time of 48 months (95% confidence interval [CI]: 36.41–59.59). High-risk patients (hazard ratio [HR]: 12.467; 95% CI: 11.029–12.951), partial response (PR) (HR: 1.200; 95% CI: 1.475–2.509) or progression disease (PD) (HR: 1.924; 95% CI: 1.623–3.012) after induction chemotherapy, and intracranial metastasis (HR: 3.057; 95% CI: 0.941–4.892) were independent risk factors for survival (*p* < 0.05) and postrelapse survival (*p* < 0.05). NBL relapse, male sex, and PR or PD after induction chemotherapy were risk factors for event-free survival (*p* < 0.05).

**Conclusions:**

In addition to previously established independent risk factors, such as age, risk group, and relapse, efficacy of induction chemotherapy and intracranial metastasis play significant roles in the prognosis of NBL.

## Background

Neuroblastoma (NBL), a malignancy of neural crest cells, is the most common, extracranial, solid tumor found in children, accounting for up to 15% of pediatric malignancy-related deaths [1]. Despite advances in NBL therapy in recent decades [2], high-risk NBL, classified according to guidelines established by the International Neuroblastoma Risk Group (INRG) [3], is a challenging cancer in children. The prognosis of children with intermediate- and high-risk NBL remains poor [4].

Recently, NBL has received considerable attention in low- and middle-income countries. Over 90% of children with NBL are diagnosed before the age of 5 years [5]. Evidence suggests that large gaps in diagnosis, treatment, and care of patients with NBL between high- and low-income countries are due to economic differences and not ethnicity. The prevalence of NBL in North America and the United Kingdom is approximately 1/650 and 1/100 live births, respectively [6, 7], whereas that in African–American children is 8.5/million live births [8, 9]. However, African–American children in the US are at a higher risk of developing NBL than European–American children [10]. Additionally, the treatment rate of high-risk NBL has been reported to be < 40% in developed countries, and the complete remission rate in patients with stage IV NBL is 18%. The estimated 5-year overall survival (OS) is approximately 60% even in patients receiving increased-intensity treatment in high-income countries [11].

However, there is limited evidence regarding the characteristics and prognosis of patients with NBL in China during the past decade. China is the largest developing country in the world, and its economy has increased considerably in recent decades, although the distribution of the pediatric care system remains uneven [12]. Therefore, the current study aimed to evaluate the potential risk factors, including economic and clinical factors, treatment strategies, and prognosis of intermediate- and high-risk NBL in China.

## Methods

### Patients

This prospective study included patients who received treatment between January 2006 and March 2015 at the Pediatric Hematology Center of Beijing Tongren Hospital, affiliated with Capital Medical University. The inclusion criteria were as follows: diagnosis of intermediate- or high-risk NBL according to the guidelines established by INRG [3]; age ≤ 14 years; and written informed consent provided by patients or their parents. Currently, the 5-year survival rate among intermediate risk group is largely improved, but the 5-year survival rate is still poor in high-risk group. Considering also that neuroblastoma is a highly heterogeneous tumor, we only included and compared the intermediate and high-risk groups.

Intermediate-risk patients were those (1) aged < 18 months with any pathological types, except for ganglio neuroma (GN) or ganglioneuroblastoma (GNB) mixed type, and without N-MYC amplification but with 11q deletion; (2) aged ≥ 18 months with GNB nodule-type or NBL histology and without N-MYC amplification but with 11q deletion; (3) aged ≥ 18 months with GNB nodule-type or NBL histology and N-MYC amplification (copy number ≤ 4) [13]; (4) aged < 18 months with N-MYC amplification (copy number ≤ 4), diploid DNA, and distant tissue metastasis; and (5) aged < 18 months with stage IV NBL and N-MYC amplification (copy number ≤ 4) but without 11q deletion or DNA ploidy abnormalities.

High-risk patients were those (1) with any histological types, except for GN or GNB mixed type, and N-MYC amplification (any age); (2) aged ≥ 18 months with GNB nodular or NB histology and N-MYC amplification; (3) aged < 18 months with distant tissue metastasis and N-MYC amplification; (4) aged ≥ 18 months with distant tissue and organ metastasis; and (5) aged < 18 months with stage IV NBL without N-MYC amplification but with 11q deletion.

All participants received a standard regular treatment and follow-up. The protocol was approved by the ethics committees of Beijing Tongren Hospital (2020-08-SFZX-HDS).

### Treatment procedures

Children were treated until December 2010 in accordance with the A3961/3973 protocol developed by the Children’s Oncology Group, and the consolidation primarily referred to intensive chemotherapy and maintenance therapy. Before year of 2010, the chemotherapy of COG A3961/3973 were used: preoperative adjuvant chemotherapy was included 1, 2, 4, 6 cycles of cyclophosphamide + bicorubicin + vincristine; in the 3rd and 5th cycles, cisplatin + etopoplatin glycosides were used. From January 1, 2011, patients were treated with 4–6 cycles of preoperative chemotherapy consisting of a topotecan and cyclophosphamide regimen, according to the Children’s Cancer Anti-Cancer Association Pediatric Hematologic Tumor Branch Neuroblastoma Cooperative Group. After surgery, patients were treated with consolidation chemotherapy, immunotherapy, or radiotherapy depending on the prognosis [3]. Children at high risk who met the indications for stem cell transplantation received pretreatment (busulfan + melphalan) combined with autologous peripheral blood stem cell transplantation (APBSCT). High-risk patients were primarily treated with immunotherapy, including a 28-day cycle in which two oral doses of 13-cis retinoic acid (160 mg/[m^2^×d]) was administered for 14 days and was discontinued for 14 days; the total treatment comprised 6–9 cycles. The dose was reduced to 80 mg/(m^2^×d) in children with intolerance, and children with serious side effects were instructed to discontinue the drug. Children who did not receive transplants were required to maintain chemotherapy for 6 months following complete remission.

Patients with intermediate-risk NBL received ≤ 12 cycles of chemotherapy comprising cyclophosphamide, topotecan, cisplatin, etoposide, and adriamycin. In contrast, patients with high-risk NBL were administered chemotherapy at 3-week intervals. All patients with NBL aged < 1 year received 6–9 cycles of chemotherapy.

Consolidation therapy included high-dose chemotherapy with carboplatin–etoposide–melphalan [14] or bulsufan–melphalan combined with APBSCT or intensive chemotherapy, irradiation, and postconsolidation therapy to control minimal residual disease. On the first day of transplantation, recombinant human granulocyte colony-stimulating factor was intravenously administered at a dose of 5–10 µg/kg per day (+ 1 day). Engraftment was confirmed based on the recovery of peripheral white blood cell counts to > 2 × 10^9^/L, granulocyte counts to > 1.5 × 10^9^/L, absolute neutrophil counts to > 0.5 × 10^9^/L, and platelet counts to > 20 × 10^9^/L, without platelet transfusions, for at least 3 consecutive days.

The treatment might have been different for those managed before 2011. Intermediate-risk patients were treated with chemotherapy combined with surgery. Chemotherapy regimens included PECV (cisplatin + vincristine + etoplatin + cyclophosphamide) and CADO (vincristine + doxorubicin + cyclophosphamide). Surgery was mainly performed to remove the primary tumor and metastatic lymph nodes in the region. Radiotherapy was also available for children aged > 3 years. Patients at high risk were primarily treated with systemic chemotherapy combined with surgery, radiotherapy, and/or peripheral blood hematopoietic stem cell transplantation and immunotherapy.

### Outcomes

OS was defined as the time from diagnosis to all-cause death or the last follow-up. NBL relapse was defined as a new disease site or 25% increase in tumor size after the initial treatment. Refractory disease was defined as tumor nonresponse to any therapy, according to the International Neuroblastoma Response Classification criteria [15]. Postrelapse OS was defined as the time from the first relapse/progression (including relapsed refractory disease) to death or the last follow-up, whichever occurred first. Event-free survival (EFS) was defined as the time from the date of diagnosis to relapse, progression, death, or the last follow-up, whichever occurred first. The last follow-up date was March 31, 2016. Progression disease (PD), partial response (PR), and complete response (CR) were defined according to RECIST and were assessed by professional physicians.

### Economic and clinical factors

Participant data, including age at diagnosis, sex, primary cancer site, clinical stage, diagnosis date, resident location, and guardian’s attitude toward NBL, were collected. Age at diagnosis was categorized into three groups: ≤18 months, 18.1 months–4 years, and > 4 years. The clinical stages were categorized as I, II, III, and IV, according to the International Neuroblastoma Staging System (INSS) [8]. Considering the regional differences in economies and medical care, we evaluated and recorded patients’ residence information in this study. Residence was categorized into three groups, according to the 2015 gross domestic product ranking in urban China as follows: developed, underdeveloped, and backward regions. The diagnosis date was divided into two groups: between January 1, 2005 and December 31, 2010, and between January 1, 2011 and March 30, 2015.

### Statistical analysis

Frequencies and percentages were calculated for categorical variables. OS, postrelapse OS, and EFS were estimated using the Kaplan–Meier method and compared using log-rank test. Univariate and multivariate Cox proportional hazards models with the stepwise method were used to identify significant risk factors related to NBL prognosis. All analyses were conducted using SPSS (IBM, version 20.0), and a two-sided *p*-value < 0.05 was considered to indicate statistical significance.

## Results

### Patient characteristics

This study included 147 patients with NBL, confirmed by a central pathologic review of tumor tissues, according to the INSS criteria. Overall, 66% (n = 97) of the patients were boys, and the ratio of boys to girls was 1.94. The mean age at diagnosis was 3.76 (± 2.83) years (median: 3 years, range: 1 month to 15.5 years), and > 63% of the patients were diagnosed before the age of 4 years. The primary tumor sites were the retroperitoneum and adrenal gland (76.2%). In total, 30 (13.6%) patients were classified as stage III, 117 (79.6%) as stage IV, and 10 (6.8%) as stage IVs, according to the INSS criteria. Further, 30 (20.4%) patients were diagnosed as intermediate-risk, and 117 (79.6%) met the criteria for high-risk disease (Table 1).

**Table 1:** General characteristics of 147 patients with neuroblastoma

Characteristics	n	Survival status		
		Deaths	Survival	*P*
		*n* = 67	*n* = 80	
Gender				0.401
Boy	97 (66.0)	43 (44.3)	54 (55.7)	
Girl	50 (34.0)	24 (48.0)	26 (52.0)	
Age at diagnosis				0.021
≤ 18 months	36 (24.5)	10 (27.8)	26 (72.2)	
18 months – 4 years	57 (38.8)	28 (49.1)	29 (50.9)	
> 4 years	54 (36.7)	29 (53.7)	25 (46.3)	
Economics regions				0.05
Development	41 (27.9)	20 (40.8)	29 (59.2)	
Underdevelopment	54 (36.7)	20 (48.8)	21 (51.2)	
Backward	52 (35.4)	18 (34.6)	34 (65.4)	
Diagnosis date				0.001
2006–2010	66 (44.9)	43 (65.2)	23 (34.9)	
2011–2015	81 (55.1)	24 (29.6)	57 (70.4)	
Primary site				0.139
Retroperitoneal- adrenal-gland	112 (76.2)	47 (42.0)	65 (58.0)	
Mediastinal area	23 (15.6)	12 (52.2)	11 (47.8)	
Other sites*	12 (8.2)	8 (66.7)	4 (33.3)	
Metastasis sites				
Bone marrow	75 (50.3)	36 (48.0)	39 (52.0)	0.332
Lung	10 (6.8)	8 (80.0)	2 (20.0)	0.026
Multi-bone	96 (65.3)	55 (57.3)	41 (42.7)	0.001
Intracranial metastasis	22 (14.9)	19 (86.4)	3 (13.6)	0.001
Liver	29 (19.7)	15 (51.7)	14 (48.3)	0.296
Distant lymph node	62 (42.2)	30 (48.4)	32 (51.6)	0.338
Spinal canal	7 (4.8)	3 (42.9)	4 (57.1)	0.498
Relapse				< 0.001
No	74 (50.3)	17 (23.0)	57 (77.0)	
Yes	73 (49.7)	50 (68.5)	23 (31.5)	
Risk-group				< 0.001
Intermediate-risk	30 (20.4)	3 (10.0)	27 (90.0)	
High-risk	117 (79.6)	64 (54.7)	53 (45.3)	
Received systematic chemotherapy				--
Yes	147 (100)	67 (45.6)	80 (54.2)	
No	0 (0)	0.00	0.00	
Received radiotherapy				0.077
Yes	17 (11.6)	11 (64.7)	6 (35.3)	
No	130 (88.4)	56 (43.1)	74 (56.9)	
Received APBSCT				0.001
Yes	35 (76.2)	25 (71.4)	10 (28.6)	
No	112 (23.8)	42 (37.5)	70 (62.5)	
Efficacy of induction chemotherapy#				0.001
CR	96 (66.2)	31 (32.3)	65 (67.7)	
PR	32 (22.1)	18 (56.3)	14 (43.8)	
PD	17 (11.7)	16 (94.1)	1 (5.9)	
NMYC status				0.262
Yes	50 (34.0)	26 (52.0)	24 (48.0)	
No	97 (66.0)	41 (42.3)	56 (57.7)	
Year for admission				< 0.001
2006–2010	66 (44.9)	43 (65.2)	23 (34.8)	
2011–2015	81 (55.1)	24 (29.6)	57 (70.4)	

* The primary site in three patients with stage IV NBL was the spinal canal

# Two children were lost to follow-up due to treatment abandonment

# Three patients with intermediate-risk NBL died due to relapse

Furthermore, 27.9%, 36.7%, and 35.4% of patients were from developed, underdeveloped, and backward regions, and 20 (40.8%), 20 (48.8%), and 30 (34.6%) patients from these regions had died by the last date of follow-up, respectively (*p* = 0.05) (Table 1). Relapse was noted in 49.7% of these patients. The mortality rates were significantly higher in patients with NBL diagnosed between 2006 and 2010 than in those diagnosed between 2011 and 2015 (65.2% vs. 29.6%, *p* = 0.001). Moreover, the mortality rates were relatively high in patients who were diagnosed between 2006 and 2010, received APBSCT, had lung or multiple bone metastases, or had intracranial metastasis (all *p*-values < 0.05).

### Prognostic analyses

Sixty-seven patients died during a median follow-up of 32.5 months, accounting for 45.6% of all patients, indicating a median OS of 48 months (95% confidence interval [CI]: 36.4–59.6 months). Among them, 51 patients died after relapse, with a postrelapse OS of 69.9%. Ninety-seven (65.9%) relapse or progression events occurred, with a median EFS of 35.5 months (95% CI: 28.65–42.35 months).

**Fig. 1:**
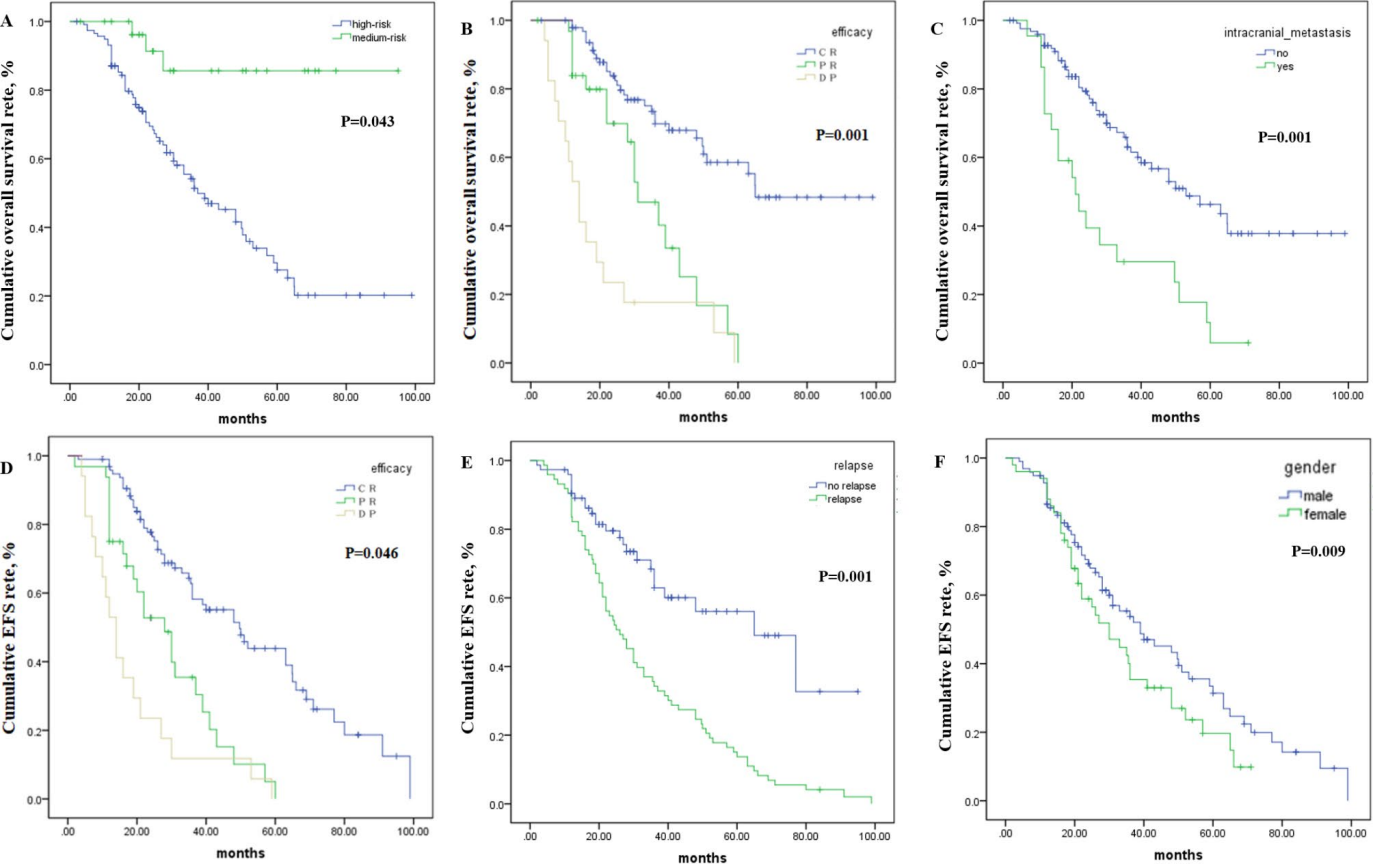
Kaplan–Meier curves of overall survival in different risk (**A**), efficacy (**B**), and intracranial metastasis (**C**) groups and event-free-survival in different efficacy (**D**), relapse (**E**), and sex (**F**) groups

Independent risk factors for OS included risk group, efficacy of induction chemotherapy, and intracranial metastasis (all *p*-values < 0.05) (Fig. 1A-C). The hazard ratio (HR) of OS was 12.467 (95% CI: 11.029–12.951) for high-risk NBL (vs. intermediate-risk NBL), 1.200 for PR (1.475–2.509) and 1.924 (1.623–3.012) for PD (vs. CR), and 3.057 (0.941–4.892) for intracranial metastasis (vs. others) (Table 2). Intracranial metastasis (HR = 2.140, 95% CI: 1.234–3.710), high-risk NBL (HR = 1.279, 95% CI: 1.182–1.849), and efficacy of induction chemotherapy (PR: HR = 1.493, 95% CI = 1.095–2.513; disease relapse: HR = 1.911, 95% CI = 1.183–6.511) were significantly associated with postrelapse OS (*p* < 0.05). Male sex (HR = 3.007, 95% CI: 1.245–4.527), relapse (HR = 2.173, 95% CI: 1.333–3.542), and efficacy of induction chemotherapy (PR: HR = 1.490, 95% CI = 1.399–2.312; disease relapse: HR = 2.270, 95% CI = 2.138–3.270) were significant risk factors for EFS (all *p* < 0.05) (Fig. 1D-F).

**Table 2:** Multivariable association of clinical and economic factors with overall survival, postrelapse overall survival, and event-free survival using Cox regression models

Characteristics	Events / n (%)	HR (95% CI)	P value
**Overall survival**	**67/147(45.6)**		0.001
Risk group			0.043
Intermediate-risk	3/30 (10.0)	1	
High-risk	64/117 (54.7)	12.467 (11.029 ~ 12.951)	
Efficacy of induction chemotherapy#			0.001
CR	31/96 (66.2)	1	
PR	18/32 (56.3)	1.200 (1.475 ~ 2.509)	
PD	16/17 (94.1)	1.924 (1.623 ~ 3.012)	
Metastasis sites			0.001
Others	48/125(38.4)	1	
Intracranial metastasis	19/22(86.4)	3.057 (0.941 ~ 4.892)	
**Post-relapse overall survival time**			
Post-relapse OS	51/73(69.9)		
Metastasis sites			0.001
Intracranial metastasis	15/16(93.8)	2.140 (1.234 ~ 3.710)	
Others	36/57(63.2)	1	
Risk group			0.021
Intermediate-risk	27/36(75.0)	1	
High-risk	24/37(64.9)	1.279 (1.182 ~ 1.849)	
Efficacy of induction chemotherapy#			
CR	22/39(56.4)	1	0.001
PR	10/15(62.5)	1.493 (1.095 ~ 2.513)	
PD	16/17(94.1)	1.911 (1.183 ~ 6.511)	
**Event-free survival**	**97/147(65.9)**		
Relapse			0.001
No	25/74(33.8)	1	
Yes	72/73(98.6)	2.173 (1.333 ~ 3.542)	
Gender			0.009
Male	60/97(61.9)	1	
Female	37/50(74.0)	3.007 (1.245 ~ 4.527)	
Efficacy of induction chemotherapy			0.046
CR	53/96(55.2)	1	
PR	25/32(78.1)	1.490 (1.399 ~ 2.312)	
PD	17/17(100.0)	2.270 (2.138 ~ 3.270)	

The outcomes within 5 years of follow-up showed similar risk factors for OS, postrelapse OS, and EFS (Table 3). The HR of OS was 1.200 (95% CI: 1.080–1.225) for high-risk NBL (vs. intermediate-risk NBL), 0.598 (0.387–0.924) for age ≤ 18 months and 1.138 (0.829–1.625) for age > 4 years (vs. 18 months–4 years), and 1.490 (1.409–2.695) for PR and 1.949 (1.490–7.112) for PD after induction chemotherapy (vs. patients with CR). High-risk NBL (HR = 5.286, 95% CI: 3.828–6.897) and diagnosis before 2011 (HR = 2.123, 95% CI: 1.486–3.215) were significantly related to postrelapse OS (*p* < 0.05). High-risk NBL (HR = 3.007, 95% CI: 1.245–4.527) was also a significant risk factor for EFS (*p* = 0.001).

**Table 3:** Multivariable association of clinical and economic factors with overall survival, postrelapse overall survival, and event-free survival within 5 years of follow-up, based on Cox regression models

Characteristics	Events / n (%)	HR (95% CI)	P value
**Overall survival**	**67/147(45.6)**		
Risk group			0.02
Intermediate-risk	3/30(10.0)	1	
High-risk	64/117(54.7)	1.200 (1.080 ~ 1.225)	
Age Group			0.021
≤ 18 months	10/36(27.8)	0.598 (0.387 ~ 0.924)	
18 months – 4 years	28/57(49.1)	1	
> 4 years	29/54(53.7)	1.138 (0.829 ~ 1.625)	
Efficacy of induction chemotherapy			0.001
CR	31/96 (66.2)	1	
PR	18/32 (56.3)	1.490 (1.409 ~ 2.695)	
PD	16/17 (94.1)	1.949 (1.490 ~ 7.112)	
**Post-relapse overall survival time**			
Risk group			0.011
Intermediate-risk	2/3(66.7)	1	
High-risk	49/70(70.0)	5.286 (3.828 ~ 6.897)	
Date of diagnosis			0.012
2006–2010	30/36(55.5)	2.123 (1.486 ~ 3.215)	
2011–2015	21/37(56.8)	1	
**Event-free survival**	**76/147(51.7)**		
Risk group			0.001
Intermediate-risk	4/30(13.3)	1	
High-risk	72/117(61.5)	3.007 (1.245 ~ 4.527)	

## Discussion

Our study including 147 patients with intermediate- or high-risk NBL in China revealed that high-risk NBL, intracranial metastasis, and PR or PD after induction chemotherapy were significant risk factors for OS, postrelapse OS, and EFS. High-risk NBL was also a significant risk factor for 5-year OS, postrelapse OS, and EFS.

NBL is the most common extracranial malignant tumor with a high metastasis rate in children. NBL has a diverse prognosis, ranging from near-uniform survival to high fatality risk [9]. The general ratio of male to female NBL diagnoses is 1.2:1 [16]. However, in our study, the ratio of male to female patients was approximately 2:1, which was relatively higher than that reported in a previous study. This finding might be partially attributable to the small sample size and single-center data. Moreover, the conventional concept of male-child preference may have affected this discrepancy.

The prognosis of patients with NBL diagnosed with stage IV or IVs and high-risk NBL is poor [17]. Timely NBL diagnosis is critical for effective therapy. In our study, > 80% of patients were classified as stages IV and IVs according to the INSS criteria, and approximately 80% of them were defined as high-risk, suggesting serious delays in diagnosis. These data indicated that health education should be promoted both among physicians and the general population to enhance the timely diagnosis and treatment of NBL.

Thus far, several advanced NBL therapies have been proposed according to different stages and risk levels [18]. However, chemotherapy resistance, such as multidrug resistance, and relapse can result in poor prognosis and low survival rates [19]. Induction chemotherapy (to reduce the tumor burden and metastases) is one of the three types of therapy for high-risk NBL. The other two therapies include combination chemotherapy with four or six agents (commonly carboplatin, cisplatin, cyclophosphamide, doxorubicin, vincristine, and topotecan) and peripheral blood stem cell harvest [2]. Drug resistance (acquired) often occurs during induction, leading to NBL relapse when tumors are not eliminated by myeloablative and maintenance therapies [20, 21]. We determined that the efficacy of induction chemotherapy, such as OS, postrelapse OS, and EFS, may be a marker of prognosis in patients with high- or intermediate-risk NBL. This indicates that if the response to induction chemotherapy is poor, as observed in patients with PD, further treatment may result in a poor prognosis, with approximately two times the risk of death or postrelapse death and 1.798 times the risk of relapse, progression, or death compared with those with CR after induction chemotherapy. Even after 5 years, PD (vs. CR) after induction chemotherapy was related to a 1.949 times higher risk of death. However, this finding should be confirmed in further well-designed and large-scale studies.

In our study, the 5-year OS and EFS rates were significantly lower in patients with high-risk NBL than in those with intermediate-risk NBL. Previous evidence also suggests that intermediate-risk NBL is associated with a longer OS and EFS than high-risk NBL [22]. The primary reason for death among high-risk patients was relapse or multidrug resistance [23–26]. The rate of relapse or death in this study was higher among high-risk patients than among intermediate-risk patients, suggesting a close relationship between the poor prognosis and high relapse rate of high-risk patients. Moreover, early diagnosis is closely associated with good outcomes and is critical for patients with NBL.

In general, NBL is characterized by early recurrence and metastasis, and some patients may experience progression during treatment. Therefore, long-term treatment and poor prognosis are crucial in NBL management. Recently, although the rate of visits has gradually increased, the proportion of outpatients in economically backward regions was significantly higher after 2011 (78.8% [41/52]) than before 2011 in the present study. Therefore, in this study, we included the date of diagnosis (before or after 2010) as a potential factor of prognosis.

Studies have indicated that immunotherapy is the primary strategy for minimal residual disease [11, 27], and clinical studies have mostly focused on monoclonal antibodies (MoAbs) against the glycolipid disialoganglioside GD2 [28]. Recently, three anti-GD2 antibodies, including murine 14G2a, human-mouse chimeric ch14.18, and 3F8, were tested in the clinic. Anti-GD2 MoAbs induce cellular cytotoxicity against NBL and are most effective as effector cells. The combination of the cytokines—interleukin-2 and granulocyte–macrophage colony-stimulating factor—and the anti-GD2 MoAb ch14.18 (dinutuximab) has resulted in significant improvements in the outcomes of high-risk NBL [29, 30]. The US Food and Drug Administration and the European Medicines Agency have recently approved dinutuximab (Unituxin®) for treating patients with high-risk NBL who achieved at least PR after multimodality therapy [31]. However, these drugs are still unavailable in developing countries due to their high costs. Therefore, using alternative methods to improve the prognosis of patients with NBL, especially in those with high-risk NBL, is important.

The current study has certain limitations. First, the sample size was relatively small, limiting the statistical power for subgroup analyses. Second, the economic factors examined in this study were limited. Third, this study included patients from a single center in China; thus, the generalizability of our conclusions might be limited. Therefore, multicenter large-scale studies on other economic factors are warranted.

## Conclusions

In conclusion, our study showed that in China, high-risk NBL, intracranial metastasis, and PR or PD after induction chemotherapy were significant risk factors for OS, postrelapse OS, and EFS. Moreover, high-risk NBL was a risk factor for 5-year OS, postrelapse OS, and EFS. These findings suggest that the long-term survival of patients with high-risk NBL and those with inadequate response to induction chemotherapy is poor because of delayed diagnosis, high relapse rates, and ineffective drugs.

## Data Availability

The datasets used and/or analysed during the current study are available from the corresponding author on reasonable request.

## Abbreviations

APBSCT: autologous peripheral blood stem cell transplantation
COG: Children’s Oncology Group
EFS: Event-free survival
G CSF: granulocyte-colony stimulating factor
GM-CSF: granulocyte-macrophage colony-stimulating factor
INRC: International Neuroblastoma Response Classification
INRG: International Neuroblastoma Risk Group
INSS: International Neuroblastoma Staging System
MoAb: monoclonal antibodies
MRD: minimal residual disease
NBL: Neuroblastoma
OS: Overall survival
PD: progressive disease
PLT: platelet
PROS: Post-relapse overall survival time
